# The prevalence of opportunistic infections and malignancies in autopsied patients with human immunodeficiency virus infection in Japan

**DOI:** 10.1186/1471-2334-14-229

**Published:** 2014-04-29

**Authors:** Harutaka Katano, Tsunekazu Hishima, Makoto Mochizuki, Yoshinori Kodama, Naoki Oyaizu, Yasunori Ota, Sohtaro Mine, Toru Igari, Atsushi Ajisawa, Katsuji Teruya, Junko Tanuma, Yoshimi Kikuchi, Tomoko Uehira, Takuma Shirasaka, Tomohiko Koibuchi, Aikichi Iwamoto, Shinichi Oka, Hideki Hasegawa, Seiji Okada, Akira Yasuoka

**Affiliations:** 1 Department of Pathology, National Institute of Infectious Diseases, 1-23-1 Toyama, Shinjuku-ku, Tokyo 162-8640, Japan; 2 Department of Pathology, Tokyo Metropolitan Komagome Hospital, 3-18-22 Honkomagome, Bunkyo-ku, Tokyo 113-8677, Japan; 3 Department of Pathology, National Center for Global Health and Medicine Hospital, 1-21-1 Toyama, Shinjuku-ku, Tokyo 162-8655, Japan; 4 Department of Pathology, Kyorin University School of Medicine, 6-20-2 Shinkawa, Mitaka City, Tokyo 181-8611, Japan; 5 Department of Pathology, Osaka National Hospital, 2-1-14 Hoenzaka, Chuo-ku, Osaka 540-0006, Japan; 6 Department of Pathology, Research Hospital, the Institute of Medical Science, the University of Tokyo, 4-6-1 Shirokanedai, Minato-ku, Tokyo 108-8639, Japan; 7 Department of Infectious Diseases, Tokyo Metropolitan Komagome Hospital, 3-18-22 Honkomagome, Bunkyo-ku, Tokyo 113-8677, Japan; 8 AIDS Clinical Center, National Center for Global Health and Medicine Hospital, 1-21-1 Toyama, Shinjuku-ku, Tokyo 162-8655, Japan; 9 Department of Infectious Diseases, Osaka National Hospital, 2-1-14 Hoenzaka, Chuo-ku, Osaka 540-0006, Japan; 10 Department of Infectious Diseases and Applied Immunology, Hospital, the Institute of Medical Science, the University of Tokyo, 4-6-1 Shirokanedai, Minato-ku, Tokyo 108-8639, Japan; 11 Division of Infectious Diseases, Advanced Clinical Research Center, the Institute of Medical Science, the University of Tokyo, 4-6-1 Shirokanedai, Minato-ku, Tokyo 108-8639, Japan; 12 Center for AIDS Research, Kumamoto University, 2-2-1 Honjo, Kumamoto 860-0811, Japan; 13 Oomura City Municipal Hospital, 133-2 Kogashima-cho, Omura City, Nagasaki 865-8561, Japan

**Keywords:** AIDS, Opportunistic infections, Autopsy, Antiretroviral therapy

## Abstract

**Background:**

Opportunistic infections and malignancies such as malignant lymphoma and Kaposi sarcoma are significant complications of human immunodeficiency virus (HIV) infection. However, following the introduction of antiretroviral therapy in Japan in 1997, the incidence of clinical complications has decreased. In the present study, autopsy cases of HIV infection in Japan were retrospectively investigated to reveal the prevalence of opportunistic infections and malignancies.

**Methods:**

A total of 225 autopsy cases of HIV infection identified at 4 Japanese hospitals from 1985–2012 were retrospectively reviewed. Clinical data were collected from patient medical records.

**Results:**

Mean CD4 counts of patients were 77.0 cells/μL in patients who received any antiretroviral therapy during their lives (ART (+) patients) and 39.6 cells/μL in naïve patients (ART (−) patients). Cytomegalovirus infection (142 cases, 63.1%) and *pneumocystis* pneumonia (66 cases, 29.3%) were the most frequent opportunistic infections, and their prevalence was significantly lower in ART (+) patients than ART (−) patients. Non-Hodgkin lymphoma and Kaposi sarcoma were observed in 30.1% and 16.2% of ART (−) patients, and 37.9% and 15.2% of ART (+) patients, respectively. Malignant lymphoma was the most frequent cause of death, followed by cytomegalovirus infection regardless of ART. Non-acquired immunodeficiency syndrome (AIDS)-defining cancers such as liver and lung cancer caused death more frequently in ART (+) patients (9.1%) than in ART (−) patients (1.5%; *P* = 0.026).

**Conclusions:**

The prevalence of infectious diseases and malignancies were revealed in autopsy cases of HIV infection in Japan. The prevalence of cytomegalovirus infection and *pneumocystis* pneumonia at autopsy were lower in ART (+) patients than ART (−) patients. Higher prevalence of non-AIDS defining malignancies among ART (+) patients than ART (−) patients suggests that onsets of various opportunistic infections and malignancies should be carefully monitored regardless of whether the patient is receiving ART.

## Background

Opportunistic infections such as *Pneumocystis jirovecii* pneumonia (PCP), cytomegalovirus (CMV), non-tuberculous mycobacteria (NTM), and fungal infections are frequently found in patients with acquired immunodeficiency syndrome (AIDS) [1]. The most frequent opportunistic infection among patients with AIDS is CMV infection, which commonly causes retinitis, pneumonia, and gastrointestinal tract ulcers. PCP is also a frequent infectious disease in the lungs of patients with AIDS. Additionally, malignancies such as non-Hodgkin lymphoma (NHL) and Kaposi sarcoma (KS) are significant complications. NHL in particular is not easily controlled and is a frequent AIDS-associated cause of death. Interestingly, KS has only been reported in homosexual patients, and patients with multifocal KS lesions have a poor prognosis.

The introduction of antiretroviral therapy (ART) has drastically changed the incidence of opportunistic infections in patients infected with human immunodeficiency virus 1 (HIV-1), resulting in a decline in mortality rates [2-7]. ART has decreased the frequencies of CMV, PCP, and NTM infections in patients with AIDS [7]; however, the frequency of NHL has not changed dramatically [8]. Additionally, non-AIDS-defining malignancies such as liver, lung, and gastric cancers have been observed in patients with AIDS, regardless of ART [9]. A recent study demonstrated that low CD4 counts at ART initiation was associated with a greater risk of KS and lymphoma, whereas other cancers increased over time with ART, likely reflecting an increased risk of cancer with aging [10], low CD4 counts, and cigarette smoking [11-13].

Although mortality rates have decreased dramatically with the use of ART, its effect in many patients with AIDS is limited, and AIDS-associated complications remain a leading cause of death [14,15]. Additionally, untreated HIV-1-positive patients with severe AIDS-defining illnesses frequently visit hospitals and often rapidly succumb to suddenly aggressive progression of their illness [16,17]. Systematic pathological analysis of autopsy cases can provide useful information related to the cause of death and the distribution of pathogens in patients. However, there have been few reports describing the prevalence of infectious diseases and malignancies in autopsied patients with HIV infection [1,18]. A previous study using samples from autopsied patients with HIV infection during 1982–1998 demonstrated the prevalence of CMV, PCP, and NTM infections decreased during the study period [18]. The same study reported that, although the prevalence of KS was unchanged, the prevalence of NHL increased during the study period [18]. To the best of our knowledge, there are no reports demonstrating changes in the prevalence of opportunistic infections in autopsy cases of HIV infection following the introduction of ART after 2000.

In the present study, autopsy cases of HIV infection in Japan were retrospectively investigated to determine the prevalence of opportunistic infections and malignancies often found in patients with AIDS, including non-AIDS-defining malignancies. Additionally, the association of ART use with the prevalence of opportunistic infections and malignancies was investigated.

### Patients and methods

#### Patients

The present study was approved by the Institutional Review Board of the National Institute of Infectious Diseases (Approval No. 356) and of four hospitals in Japan: Tokyo Metropolitan Komagome Hospital, National Center for Global Health and Medicine, Research Hospital, the Institute of Medical Science, the University of Tokyo, and Osaka National Hospital. Each hospital enrolled in the present study is a central hospital for AIDS treatment in Tokyo and Osaka, and has performed more than 15 autopsies of patients infected with HIV. According to a national autopsy survey by the Japan Pathology Society, 828 patients infected with HIV were autopsied in Japan from 1987–2009. During the period 1985–2009, 215 patients infected with HIV were autopsied at the 4 aforementioned hospitals. Thus, the number of cases in this study covered approximately 26% of all autopsied HIV cases. Ten cases autopsied in the period 2010–2012 were added to the 215 cases, making a total of 225 patients analyzed in this study (Table 1), of which 95.1% were male. The patients’ ages at death ranged from 12 to 80 years, with a mean age of 44.4 years (median 44 years). Among them, 35.6% were homosexual, and 29.3% received ART (Table 1). The mean CD4 count at the last blood examination before death was 51.5 cells/μL (range: 0–560 cells/μL; median: 13.5 cells/μL). ART was introduced in Japan in 1997. In this study, ART was defined as any combination of therapy that included two nucleoside or nucleotide reverse transcriptase inhibitors plus a non-nucleoside reverse transcriptase inhibitor, protease inhibitor, or abacavir (another nucleotide reverse transcriptase inhibitor). Additionally, ART (+) patients were defined as patients who received any ART during their lifetime, whereas ART (−) patients were as patients who did not receive ART.

**Table 1:** Characteristics of the patients infected with HIV

**Factors**	**Groupings**	**Total patients**		**ART (−) patients**		**ART (+) patients**		***P *value**
		**n**	**%**	**n**	**%**	**n**	**%**	
Total		225*	100%	136	100%	66	100%	
Sex	Male	214	95.1%	128	94.1%	63	95.5%	0.695**
	Female	11	4.9%	8	5.9%	3	4.5%	
Age at death	<10 years	0	0.0%	0	0.0%	0	0.0%	0.028***
	11–20	2	0.9%	2	1.5%	0	0.0%	
	21–30	30	13.3%	22	16.2%	6	9.1%	
	31–40	60	26.7%	34	25.0%	19	28.8%	
	41–50	69	30.7%	48	35.3%	14	21.2%	
	51–60	34	15.1%	18	13.2%	14	21.2%	
	61–70	24	10.7%	10	7.4%	10	15.2%	
	71–80	5	2.2%	1	0.7%	3	4.5%	
	>81	0	0.0%	0	0.0%	0	0.0%	
	Unknown	1	0.4%	1	0.7%	0	0.0%	
Risk factor	Homosexual	80	35.6%	52	38.2%	24	36.4%	0.800**
	Heterosexual	38	16.9%	24	17.6%	10	15.2%	
	Blood product	37	16.4%	29	21.3%	7	10.6%	
	Other	9	4.0%	5	3.7%	4	6.1%	
	Unknown	61	27.1%	26	19.1%	21	31.8%	
CD4 count before death	<50 cells/μL	122	54.2%	80	58.8%	33	50.0%	0.639***
	51–100	25	11.1%	11	8.1%	14	21.2%	
	101–200	13	5.8%	4	2.9%	9	13.6%	
	201–300	5	2.2%	5	3.7%	0	0.0%	
	301–400	3	1.3%	1	0.7%	2	3.0%	
	>401	4	1.8%	1	0.7%	3	4.5%	
	Unknown	53	23.6%	34	25.0%	5	7.6%	

ART, antiretroviral therapy. ART (+) patients were defined as patients who received any ART during their lifetime, whereas ART (−) patients were as patients who did not receive any ART in their lifetime.

*Total number of patients = 225 and included 23 patients with unknown ART status.

**P values were calculated for the rates of male or homosexuals between ART (−) and (+) patients by Chi-square test.

***P values were calculated for age or CD4 counts between all ART (−) and (+) patients by Mann–Whitney *U*-test. Bold font indicates statistical significance.

## Methods

Pathological findings were collected from autopsy records. CMV infection was determined by the infiltration of large cells with typical inclusion bodies. Infections by other viral agents such as hepatitis B virus, herpes simplex virus, hepatitis C virus, JC virus (causing progressive multifocal leukoencephalopathy), and varicella zoster virus were confirmed by immunohistochemistry or polymerase chain reaction. HIV encephalopathy was defined by morphological features indicating the presence of syncytial giant cells and detection of HIV-1 antigen by immunohistochemistry in the brain. Bacterial infection was identified by Gram stain, and in some cases, species of bacteria were identified by bacterial cultures. Tuberculosis and NTM infection were determined by acid-fast stain and/or PCR. Fungal and protozoan infections such as PCP, toxoplasma, *Candida*, *Aspergillus*, and *Cryptococcus* infection, were determined morphologically using Grocott’s methenamine silver stain, periodic acid-Schiff stain, or/and immunohistochemistry. The histological sub-typing of malignant lymphoma was based on the World Health Organization classification, fourth edition. KS was confirmed by immunohistochemistry for Kaposi sarcoma-associated herpesvirus-encoded latency-associated nuclear antigen 1. Causes of death were determined by pathologists at each hospital based on the severity, distribution, and type of illness in the pathological findings of autopsy. Clinical data, such as age at autopsy, sex, risk factors, CD4 cell counts at the last blood examination before death, and use of ART in their lifetime were collected from medical records. Analysis of statistical significance was carried out using Mann–Whitney *U*-test for non-parametric two-sample analysis and Chi-squared test for contingency table analysis.

## Results

**Figure 1:**
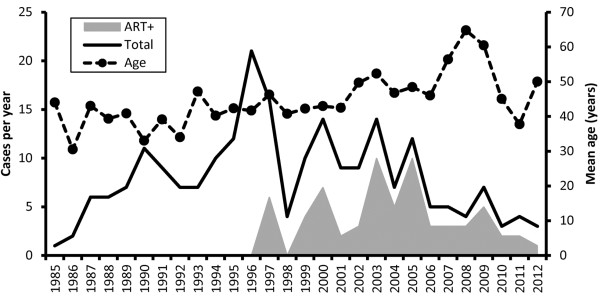
**Annual number and mean age of AIDS-related autopsies.** The solid line indicates total number of AIDS autopsies in each year. The gray area indicates the number of patients on ART in these autopsy cases. The broken bar indicates the mean age.

After the introduction of ART in Japan in 1997, the total number of autopsies conducted on patients with HIV infection has slowly decreased whereas the mean age at autopsy has increased slightly (Figure 1). After 1997, 66 of 126 patients (52.6%) received ART during their lifetime. The mean age at death of patients on ART was 47.3 years, which was significantly higher than that of ART naïve patients (42.6 years; *P* = 0.028; Mann–Whitney *U*-test). Mean CD4 counts of ART (−) and (+) patients at the last blood examination before death were not significantly different (39.6 and 77.0 cells/μL, respectively, *P* = 0.63, Mann–Whitney *U*-test).

**Figure 2:**
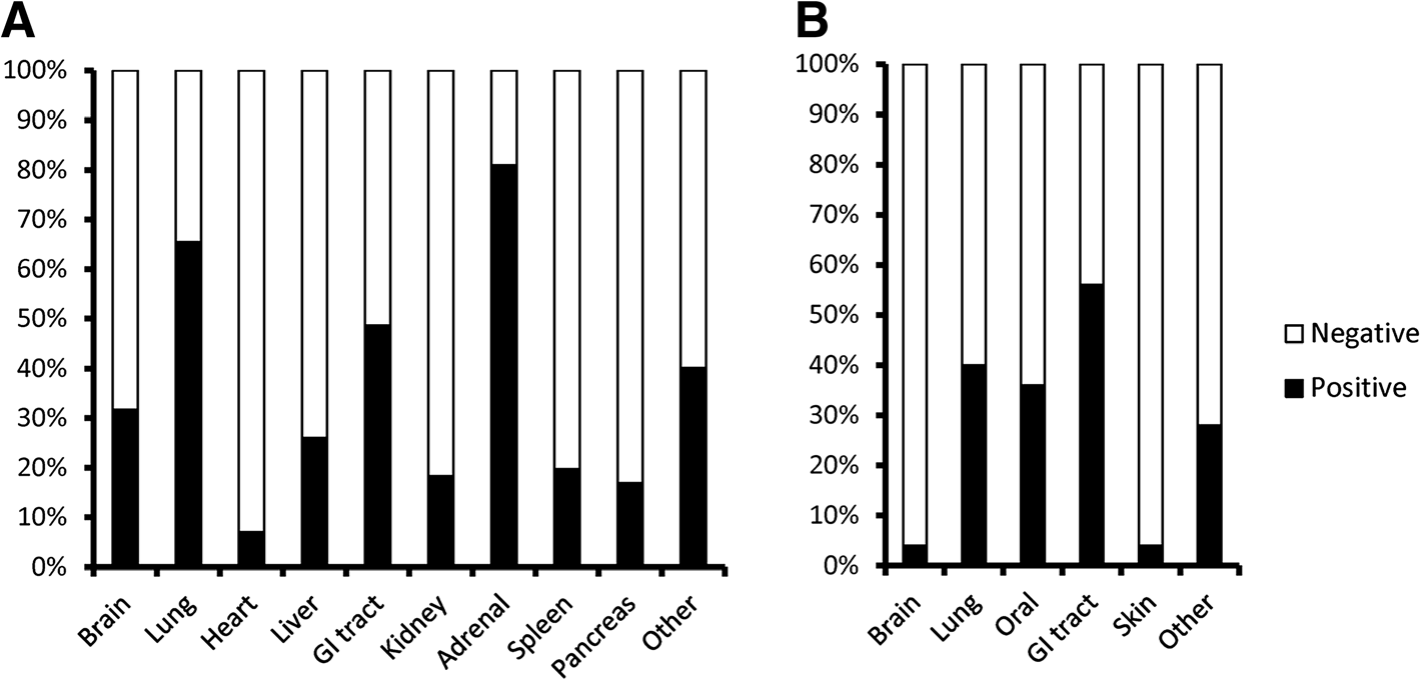
**Distribution of cytomegalovirus and *Candida albicans*. (A)** CMV positive rate in each organ. Black bar indicates the CMV positive rate in each organ from 142 CMV-positive patients. Because CMV was detected in more than one organ per patient, the sum of the black bars is over 100%. **(B)** The positive rate of *Candida albicans* in each organ. Black bar indicates the positive rate of *Candida albicans* per organ from 25 *Candida albicans*-positive patients.

CMV was the most commonly identified pathogen among the autopsy cases (Table 2) and was detected in various organs, the most frequent being the adrenal gland (Figure 2A). PCP and NTM were also common pathogens found in the lungs of autopsied patients. *Candida albicans* was frequently detected in the gastrointestinal tract and oral cavity (Figure 2B). The prevalence of CMV and PCP was significantly lower in ART (+) patients than in ART (−) patients (Table 2). There was no significant difference in the prevalence of other opportunistic infections such as NTM and *Candida* or prevalence of HIV encephalopathy between ART (+) and (−) patients (Table 2).

**Table 2:** Infectious diseases and malignancies in AIDS-associated autopsies

	**All patients**		**ART (−) patients**		**ART (+) patients**			
	**n**	**%**	**n**	**%**	**n**	**%**	***P *values**	
Total	225	100.0%	136	100.0%	66	100.0%		
Infectious diseases								
Cytomegalovirus	142	63.1%	97	71.3%	25	37.9%	**<0.001**	
*Pneumocystis jirovecii* pneumonia	66	29.3%	43	31.6%	11	16.7%	**0.024**	
Non-tuberculous mycobacterium	31	13.8%	20	14.7%	8	12.1%	0.618	
*Candida*	25	11.1%	17	12.5%	6	9.1%	0.474	
*Aspergillus*	24	10.7%	17	12.5%	4	6.1%	0.160	
Human immunodeficiency virus encephalopathy	21	9.3%	13	9.6%	6	9.1%	0.915	
*Cryptococcus*	16	7.1%	11	8.1%	3	4.5%	0.526	Y
Hepatitis B virus	12	5.3%	6	4.4%	5	7.6%	0.549	Y
Herpes simplex virus	12	5.3%	1	0.7%	1	1.5%	0.816	Y
Toxoplasmosis	11	4.9%	9	6.6%	3	4.5%	0.789	Y
Hepatitis C virus	9	4.0%	3	2.2%	5	7.6%	0.147	Y
Progressive multifocal leukoencephalopathy	8	3.6%	4	2.9%	2	3.0%	0.684	Y
Tuberculosis	6	2.7%	4	2.9%	0	0.0%	0.385	Y
Varicella zoster virus	4	1.8%	2	1.5%	2	3.0%	0.835	Y
Multicentric Castleman disease	2	0.9%	1	0.7%	1	1.5%	0.816	Y
Malignancies								
Non Hodgkin lymphoma	71	31.6%	41	30.1%	25	37.9%	0.272	
Kaposi sarcoma	38	16.9%	22	16.2%	10	15.2%	0.852	
Endocervical cancer	0	0.0%	0	0.0%	0	0.0%	–	
Non-AIDS defining malignancies	20	8.9%	10	7.4%	10	15.2%	0.082	
Hepatic cancer	8	3.6%	4	2.9%	4	6.1%	0.495	Y
Lung cancer	6	2.7%	2	1.5%	4	6.1%	0.174	Y
Leukemia	2	0.9%	0	0.0%	2	3.0%	0.200	Y
Hodgkin lymphoma	2	0.9%	1	0.7%	1	1.5%	0.816	Y
Gastric cancer	1	0.4%	1	0.7%	0	0.0%	0.711	Y
Other cancer	3	1.3%	3	2.2%	0	0.0%	0.551	Y

*P* values were calculated by Chi-square test. Y indicates the use of Chi-square test with Yates correction. Bold font indicates statistical significance. ART, antiretroviral therapy. Because more than one illness was detected in patients, the numbers of all illness are greater than the total number.

Malignancies were identified in 50.2% (113/225) of all cases (Table 2). NHL was the most frequent malignancy with a lower prevalence in ART (−) patients (30.1%) than ART (+) patients (37.9%); however, the difference was not significant (Table 2). Diffuse large B-cell lymphoma was the most frequent histological subtype of NHL followed by Burkitt lymphoma, primary effusion lymphoma, and plasmablastic lymphoma (Table 3). Epstein–Barr virus positivity in lymphoma cases was significantly lower in ART (+) patients compared with ART (−) patients (*P* = 0.001, Chi-square test). KS was frequently found in the skin as well as other sites such as the gastrointestinal tract upon autopsy. In addition to NHL and KS, non-AIDS-defining malignancies such as Hodgkin lymphoma (HL), hepatic cancer, lung cancer, and leukemia were also observed in 20 patients. The prevalence of non-AIDS-defining malignancies was higher in ART (+) patients compared with ART (−) patients (Table 2).

**Table 3:** Non-Hodgkin lymphoma and Kaposi sarcoma in AIDS-associated autopsies

		**Total**		**ART (−) patients**		**ART (+) patients**		***P *values**	
		**n**	**%**	**n**	**%**	**n**	**%**		
All NHL cases		71	100.0%	41	100.0%	25	100.0%		
Histology	DLBCL	53	74.6%	30	73.2%	18	72.0%	0.917	
	BL	4	5.6%	3	7.3%	1	4.0%	0.987	Y
	PEL	5	7.0%	4	9.8%	1	4.0%	0.706	Y
	PBL	1	1.4%	1	2.4%	0	0.0%	0.801	Y
	Other	6	8.5%	2	4.9%	4	16.0%	0.279	Y
	Unknown	2	2.8%	1	2.4%	1	4.0%	0.703	Y
Site	Nodular	1	1.4%	0	0.0%	1	4.0%	0.801	Y
	Extranodular	45	63.4%	28	68.3%	12	48.0%	0.102	
	Both	21	29.6%	11	26.8%	10	40.0%	0.265	
	Unknown	4	5.6%	2	4.9%	2	8.0%	0.987	Y
PCNS	Yes	27	38.0%	18	43.9%	6	24.0%	0.103	
EBV	Positive	52	73.2%	35	85.4%	12	48.0%	**0.001**	
KSHV	Positive	6	8.5%	5	12.2%	1	4.0%	0.495	Y
Cause of death	Yes	50	70.4%	33	80.5%	16	64.0%	0.137	
All KS cases		38		22		10			
Site	Skin	32	84.2%	19	86.4%	9	90.0%	0.410	
	GI tract	27	71.1%	15	68.2%	8	80.0%	0.705	
	Lung	21	55.3%	11	50.0%	6	60.0%	0.799	
	Lymph node	20	52.6%	13	59.1%	6	60.0%	0.502	
	Other	16	42.1%	0	0.0%	0	0.0%	0.787	Y
Cause of death	Yes	11	29.0%	7	31.8%	2	20.0%	0.791	Y

NHL, Non-Hodgkin lymphoma; DLBCL, diffuse large B-cell lymphoma; BL, Burkitt lymphoma; PEL, primary effusion lymphoma; PBL, plasmablastic lymphoma; PCNS, primary central nervous system lymphoma; EBV, Epstein–Barr virus; KSHV, Kaposi sarcoma-associated herpes virus; KS, Kaposi sarcoma; GI, gastrointestinal. *P* values were calculated by Chi-square test. Y indicates the use of Chi-square test with Yates correction. Bold font indicates statistical significance.

The lung was the most frequent target for pathogens in patients with AIDS and 173 (76.9%) autopsy cases demonstrated the presence of lung-related illnesses (Table 4), which were significantly more frequent in ART (−) patients (112/136, 82.4%) than ART (+) patients (42/66, 63.6%) (*P* = 0.003, Chi-square test). CMV then PCP was the most frequently observed lung-related illnesses. The brain was the second most frequently affected organ in the autopsy cases. Although the brain was not investigated in 53 autopsies, 85 of the remaining 172 cases (49.4%) had brain-related illnesses, with CMV infection the most common, followed by lymphoma and HIV encephalopathy (Table 5). However, there was no significant difference in the rate of brain-related illnesses in ART (+) (37.8%, 17 of 45) or ART (−) patients (52.7%, 58/110) (*P* = 0.091, Chi-square test).

**Table 4:** Lung disease in patients infected with HIV

**Illness**	**n**	**% of total patients**
		**(n = 225)**
Any illness	173	76.9%
Cytomegalovirus infection	93	41.3%
*Pneumocystis jirovecii* pneumonia	66	29.3%
Any bacterial pneumonia	31	13.8%
*Aspergillus* infection	23	10.2%
Kaposi sarcoma	21	9.3%
Non-tuberculous mycobacterium infection	14	6.2%
*Cryptococcosis*	11	4.9%
*Candida* infection	10	4.4%
Tuberculosis	4	1.8%

Because more than one illness was detected in patients, the numbers of all illness are greater than the total number.

**Table 5:** Brain disease in patients infected with HIV

**Illness**	**n**	**% in total autopsied brains**
		**(n = 172)**
Any illness	85	49.4%
Cytomegalovirus infection	45	26.1%
Malignant lymphoma	26	15.1%
HIV encephalopathy	21	12.2%
Progressive multifocal leukoencephalopathy	8	4.7%
Toxoplasmosis	8	4.7%
Non-tuberculous mycobacterium infection	4	2.3%
*Aspergillus* infection	2	1.2%
Varicella zoster virus infection	2	1.2%
Herpes simplex virus infection	1	0.6%
Glioblastoma	1	0.6%
*Candida* infection	1	0.6%

Because more than one illness was detected in patients, the numbers of all illness are greater than the total number.

We also investigated the direct causes of death in the autopsied patients (Table 6). Lymphoma was the most frequent cause of death, followed by CMV infection. Non AIDS-defining cancers as a cause of death were significantly different between ART (−) (2, 1.5%) and ART (+) patients (6, 9.1%) (*P* = 0.026; Chi-square test with Yates correction). The prevalence of CMV, pneumonia, PCP, and NTM as a cause of death were lower in ART (+) patients compared with ART (−) patients, but no significant differences were observed between the groups.

**Table 6:** Cause of death in AIDS-associated autopsies

	**All**		**ART (−) patients**		**ART (+) patients**			
	**n**	**%**	**n**	**%**	**n**	**%**	***P *** **values**	
Total*	225	100.0%	136	100.0%	66	100.0%		
Malignant lymphoma	50	22.2%	33	24.3%	16	24.2%	0.997	
Cytomegalovirus	44	19.6%	27	19.9%	9	13.6%	0.279	
Pneumonia	31	13.8%	19	14.0%	9	13.6%	0.949	
*Pneumocystis jirovecii* pneumonia	30	13.3%	21	15.4%	4	6.1%	0.058	
Non-tuberculous mycobacterium	12	5.3%	10	7.4%	2	3.0%	0.367	Y
Kaposi sarcoma	11	4.9%	7	5.1%	2	3.0%	0.749	Y
Progressive multifocal leukoencephalopathy	8	3.6%	4	2.9%	2	3.0%	0.684	Y
Cancer	8	3.6%	2	1.5%	6	9.1%	**0.026**	Y
Hepatitis	8	3.6%	3	2.2%	4	6.1%	0.320	Y
*Cryptococcus*	7	3.1%	6	4.4%	0	0.0%	0.197	Y
Kidney failure	7	3.1%	4	2.9%	3	4.5%	0.861	Y
HIV encephalopathy	7	3.1%	5	3.7%	2	3.0%	0.861	Y
*Aspergillus*	6	2.7%	5	3.7%	0	0.0%	0.274	Y
Toxoplasmosis	4	1.8%	3	2.2%	1	1.5%	0.835	Y
Tuberculosis	3	0.9%	2	1.5%	0	0.0%	0.816	Y
Sepsis	3	1.3%	2	1.5%	1	1.5%	0.551	Y
*Candida*	3	1.3%	2	1.5%	0	0.0%	0.816	Y
Varicella zoster virus	2	1.3%	1	0.7%	1	1.5%	0.816	Y
*Nocardia*	1	0.4%	1	0.7%	0	0.0%	0.711	Y
Histoplasma	1	0.4%	1	0.7%	0	0.0%	0.711	Y

*Because more than one illness was detected in patients, the numbers of all illness are greater than the total number.

HIV, human immunodeficiency virus. *P* values were calculated with the Chi-square test. Y indicates the use of Chi-square test with Yates correction. Bold font indicates statistical significance.

## Discussion

In the present study, we measured the prevalence of infectious disease and malignancy in autopsy cases of HIV-infected patients identified from 1985–2012 at four central hospitals in Japan. CMV infection, PCP, NTM infection, NHL, and KS were frequently observed in the autopsy cases. The prevalence of CMV and PCP was lower in ART (−) patients compared with ART (+) patients. The prevalence of non-AIDS defining malignancies was higher among ART (+) patients than ART (−) patients, suggesting that the onset of various opportunistic infections and malignancies should be carefully monitored regardless of whether the patient is receiving ART.

The autopsy cases in the present study were predominantly male (95.1%, Table 1). Additionally, more than 70% of the autopsy cases in the present study had a CD4 count < 200 cells/μL at the last blood examination before death (Table 1). A recent clinical study demonstrated the incidence of AIDS-defining illnesses in patients with HIV infection was decreased by the introduction of ART, especially in patients with CD4 counts >200 cells/μL [2]. Thus, our findings at autopsy cannot be compared with previous clinical studies because many clinical study patients had a high range of CD4 counts and ART responses. Interestingly, there was no significant difference in the cause of death between ART (+) and (−) patients, with the exception of those with cancer (Table 6), indicating the prevalence of lethal illness did not differ between ART (+) and (−) patients.

Malignancies were frequent causes of death in the present study regardless of ART status (Table 6). Several studies demonstrated that the introduction of ART reduced the incidence of NHL in patients with HIV infection [13,15,19-23]. The use of ART has also been associated with a decrease in the incidence of KS [15,24,25]. However, an association between the incidence of non-AIDS-defining cancers and ART remains controversial. An increase of non-AIDS-defining cancers in patients receiving ART was shown in previous clinical reports [26,27], but a separate study showed that, with the exception of long-term protease inhibitor usage, ART exposure was generally not associated with a risk of non-AIDS-defining cancers [28]. The reasons for increased risk of non-AIDS-defining cancers in patients on ART are unclear, but might reflect the concomitant increase of the mean age at autopsy during the study period. This suggests that life extension of HIV-infected patients by ART results in the increased chance of developing non-AIDS events and malignancies. It was also demonstrated that ART introduction changed the pathological features of lymphoma; for example, a decrease of Epstein–Barr virus-positive lymphoma in Japanese patients with AIDS was reported [29]. Although HL was rare in the general Japanese population compared with European countries and the United States [30], the incidence of HL increased in Japanese patients on ART [17]. Thus, the increased risk of malignancies during the clinical course of HIV infection in patients receiving ART was reflected as a cause of death in the autopsy cases used in our study.

The prevalence of opportunistic infections differs among various regions and countries. In sub-Saharan African countries, more than 80% of HIV-positive patients die of infectious diseases, with disseminated tuberculosis being the most common (36%) [31]. Furthermore, there was no difference in the type of disease HIV patients succumbed to, regardless of ART status. In the USA and European countries, tuberculosis/NTM represented <10% of mortality in autopsy cases after 1996 [18]. In this study, tuberculosis was detected in only 2.7% of Japanese autopsy cases, but was the cause of death for 50% of afflicted patients. Mortality by PCP has decreased worldwide in patients with AIDS owing to prophylactic administration of an anti-PCP drug [16]. PCP was found in 36.4% (36/99 cases) of patients with AIDS before 1997, but was significantly reduced after 1997 (23.8%; 30/126 cases; *P* = 0.04; Chi-square test). This suggests that the decrease in PCP cases is associated with ART and anti-PCP prophylaxis.

Our study had several limitations. Bacterial culture was not available in this study owing to the use of formalin-fixed paraffin-embedded samples, and it was therefore difficult to identify the bacterial species responsible for many cases of pneumonia. Additionally, clinical information was limited. Information on HIV-RNA, an important indicator of ART effects, was not available for these patients. In addition, information regarding CD4 counts and the type, duration and possible interruption of ART were not available for a subset of patients. Therefore, we could not identify cases of immune reconstitution syndrome. Age at seroconversion and time living with HIV are also major predictors of HIV disease progression, however information of these parameters was limited. Thus, it should be noted that the conclusions in this study cannot be generally applied to the current HIV positive population in Japan. Furthermore, all findings in this study were obtained from autopsies.

## Conclusions

Although further studies are required to demonstrate the association between ART and illness identified at autopsy, the present study demonstrates the prevalence of infectious diseases and malignancies in autopsy cases of HIV infection in Japan. While the prevalence of CMV infection and PCP at autopsy were lower in ART (+) patients than ART (−) patients, non-AIDS–defining malignancies were observed as a cause of death more frequently in ART (+) patients than ART (−) patients.

## Abbreviations

HIV: Human immunodeficiency virus; ART: Antiretroviral therapy; AIDS: Acquired immunodeficiency syndrome; PCP: *Pneumocystis jirovecii* pneumonia; CMV: Cytomegalovirus; NTM: Non-tuberculous mycobacteria; NHL: Non-Hodgkin lymphoma; KS: Kaposi sarcoma.

## Competing interests

The authors declare no conflicts of interests.

## Authors’ contributions

HK, S Okada and AY conceived this study; TH, MM, YKod, NO, YO, SM, TI, HH, and HK performed the autopsies, pathological analyses and reviews; AA, KT, JT, YKi, TU, TS, TK, AI, and S Oka collected clinical data; HK analyzed the data, performed statistical analyses, and drafted the manuscript. All authors read and approved the final manuscript.

## Pre-publication history

The pre-publication history for this paper can be accessed here:

http://www.biomedcentral.com/1471-2334/14/229/prepub

## References

[B1] Bruno R Sacchi P Filice G Overview on the incidence and the characteristics of HIV-related opportunistic infections and neoplasms of the heart: impact of highly active antiretroviral therapy AIDS 2003 14 Suppl 1 S83 S87 12870535 10.1097/00002030-200304001-00012

[B2] Mocroft A Furrer HJ Miro JM Reiss P Mussini C Kirk O Abgrall S Ayayi S Bartmeyer B Braun D Castagna A d'Arminio Monforte A Gazzard B Gutierrez F Hurtado I Jansen K Meyer L Munoz P Obel N Soler-Palacin P Papadopoulos A Raffi F Ramos JT Rockstroh JK Salmon D Torti C Warszawski J de Wit S Zangerle R Fabre-Colin C The Incidence of AIDS-Defining Illnesses at a Current CD4 Count > =200 Cells/μL in the Post-Combination Antiretroviral Therapy Era Clin Infect Dis 2013 14 1038 1047 10.1093/cid/cit423 23921881

[B3] Mocroft A Katlama C Johnson AM Pradier C Antunes F Mulcahy F Chiesi A Phillips AN Kirk O Lundgren JD AIDS across Europe, 1994–98: the EuroSIDA study Lancet 2000 14 291 296 10.1016/S0140-6736(00)02504-6 11071184

[B4] Palella FJ Jr Delaney KM Moorman AC Loveless MO Fuhrer J Satten GA Aschman DJ Holmberg SD Declining morbidity and mortality among patients with advanced human immunodeficiency virus infection. HIV Outpatient Study Investigators N Engl J Med 1998 14 853 860 10.1056/NEJM199803263381301 9516219

[B5] Egger M Hirschel B Francioli P Sudre P Wirz M Flepp M Rickenbach M Malinverni R Vernazza P Battegay M Impact of new antiretroviral combination therapies in HIV infected patients in Switzerland: prospective multicentre study. Swiss HIV Cohort Study BMJ 1997 14 1194 1199 10.1136/bmj.315.7117.1194 9393221 PMC2127760

[B6] Young J Psichogiou M Meyer L Ayayi S Grabar S Raffi F Reiss P Gazzard B Sharland M Gutierrez F Obel N Kirk O Miro JM Furrer H Castagna A De Wit S Munoz J Kjaer J Grarup J Chene G Bucher H CD4 cell count and the risk of AIDS or death in HIV-Infected adults on combination antiretroviral therapy with a suppressed viral load: a longitudinal cohort study from COHERE PLoS Med 2012 14 e1001194 10.1371/journal.pmed.1001194 22448150 PMC3308938

[B7] Buchacz K Baker RK Palella FJ Jr Chmiel JS Lichtenstein KA Novak RM Wood KC Brooks JT HOPS-Investigators AIDS-defining opportunistic illnesses in US patients, 1994–2007: a cohort study AIDS 2010 14 1549 1559 10.1097/QAD.0b013e32833a3967 20502317

[B8] Ives NJ Gazzard BG Easterbrook PJ The changing pattern of AIDS-defining illnesses with the introduction of highly active antiretroviral therapy (HAART) in a London clinic J Infect 2001 14 134 139 10.1053/jinf.2001.0810 11531320

[B9] Silverberg MJ Chao C Leyden WA Xu L Tang B Horberg MA Klein D Quesenberry CP Jr Towner WJ Abrams DI HIV infection and the risk of cancers with and without a known infectious cause AIDS 2009 14 2337 2345 10.1097/QAD.0b013e3283319184 19741479 PMC2863991

[B10] Yanik EL Napravnik S Cole SR Achenbach CJ Gopal S Olshan A Dittmer DP Kitahata MM Mugavero MJ Saag M Moore RD Mayer K Mathews WC Hunt PW Rodriguez B Eron JJ Incidence and timing of cancer in HIV-infected individuals following initiation of combination antiretroviral Therapy Clin Infect Dis 2013 14 756 764 10.1093/cid/cit369 23735330 PMC3739467

[B11] Guiguet M Boue F Cadranel J Lang JM Rosenthal E Costagliola D Effect of immunodeficiency, HIV viral load, and antiretroviral therapy on the risk of individual malignancies (FHDH-ANRS CO4): a prospective cohort study Lancet Oncol 2009 14 1152 1159 10.1016/S1470-2045(09)70282-7 19818686

[B12] Reekie J Kosa C Engsig F Monforte A Wiercinska-Drapalo A Domingo P Antunes F Clumeck N Kirk O Lundgren JD Mocroft A Relationship between current level of immunodeficiency and non-acquired immunodeficiency syndrome-defining malignancies Cancer 2010 14 5306 5315 10.1002/cncr.25311 20661911

[B13] Clifford GM Polesel J Rickenbach M Dal Maso L Keiser O Kofler A Rapiti E Levi F Jundt G Fisch T Bordoni A De Weck D Franceschi S Cancer risk in the Swiss HIV Cohort Study: associations with immunodeficiency, smoking, and highly active antiretroviral therapy J Natl Cancer Inst 2005 14 425 432 10.1093/jnci/dji072 15770006

[B14] Lim P-L Zhou J Ditangco R Law M Sirisanthana T Kumarasamy N Chen Y-M Phanuphak P Lee CKC Saphonn V Oka S Zhang F Choi J Pujari S Kamarulzaman A Li PCK Merati T Yunihastuti E Messerschmidt L Sungkanuparph S Failure to prescribe pneumocystis prophylaxis is associated with increased mortality, even in the cART era: results from the Treat Asia HIV observational database J Int AIDS Soc 2012 14 1 1 10.1186/1758-2652-15-1 22281054 PMC3354658

[B15] Yotsumoto M Hagiwara S Ajisawa A Tanuma J Uehira T Nagai H Fujikawa Y Maeda S Kitano K Arima N Uno K Iwai T Hongo I Ota Y Fukutake K Okada S Clinical characteristics of human immunodeficiency virus-associated Hodgkin lymphoma patients in Japan Int J Hematol 2012 14 247 253 10.1007/s12185-012-1127-5 22752537

[B16] Hashimoto S Matsumoto T Nagai M Matsuyama Y Nakamura Y Umeda T Kamakura M Ichikawa S Kimura S Fukutomi K Kihara M Delays and continuation of hospital visits among HIV-infected persons and AIDS cases in Japan J Epidemiol 2000 14 65 70 10.2188/jea.10.65 10695263

[B17] Nakamura H Teruya K Takano M Tsukada K Tanuma J Yazaki H Honda H Honda M Gatanaga H Kikuchi Y Oka S Clinical symptoms and courses of primary HIV-1 infection in recent years in Japan Intern Med 2011 14 95 101 10.2169/internalmedicine.50.4137 21245631

[B18] Masliah E DeTeresa RM Mallory ME Hansen LA Changes in pathological findings at autopsy in AIDS cases for the last 15 years AIDS 2000 14 69 74 10.1097/00002030-200001070-00008 10714569

[B19] Tirelli U Spina M Gaidano G Vaccher E Franceschi S Carbone A Epidemiological, biological and clinical features of HIV-related lymphomas in the era of highly active antiretroviral therapy AIDS 2000 14 1675 1688 10.1097/00002030-200008180-00001 10985303

[B20] Kirk O Pedersen C Cozzi-Lepri A Antunes F Miller V Gatell JM Katlama C Lazzarin A Skinhoj P Barton SE Non-Hodgkin lymphoma in HIV-infected patients in the era of highly active antiretroviral therapy Blood 2001 14 3406 3412 10.1182/blood.V98.12.3406 11719381

[B21] Carrieri MP Pradier C Piselli P Piche M Rosenthal E Heudier P Durant J Serraino D Reduced incidence of Kaposi’s sarcoma and of systemic non-hodgkin’s lymphoma in HIV-infected individuals treated with highly active antiretroviral therapy Int J Cancer 2003 14 142 144 10.1002/ijc.10790 12455069

[B22] Vilchez RA Jorgensen JL Kroll MH Systemic non-Hodgkin lymphoma in HIV-infected patients in the era of highly active antiretroviral therapy Blood 2002 14 4250 4251 10.1182/blood-2002-01-0078 12043698

[B23] Wolf T Brodt HR Fichtlscherer S Mantzsch K Hoelzer D Helm EB Mitrou PS Chow KU Changing incidence and prognostic factors of survival in AIDS-related non-Hodgkin’s lymphoma in the era of highly active antiretroviral therapy (HAART) Leuk Lymphoma 2005 14 207 215 10.1080/10428190400015733 15621803

[B24] Pipkin S Scheer S Okeigwe I Schwarcz S Harris DH Hessol NA The effect of HAART and calendar period on Kaposi’s sarcoma and non-Hodgkin lymphoma: results of a match between an AIDS and cancer registry AIDS 2011 14 463 471 10.1097/QAD.0b013e32834344e6 21139489 PMC3089985

[B25] Franceschi S Lise M Clifford GM Rickenbach M Levi F Maspoli M Bouchardy C Dehler S Jundt G Ess S Bordoni A Konzelmann I Frick H Dal Maso L Elzi L Furrer H Calmy A Cavassini M Ledergerber B Keiser O Changing patterns of cancer incidence in the early- and late-HAART periods: the Swiss HIV Cohort Study Br J Cancer 2010 14 416 422 10.1038/sj.bjc.6605756 20588274 PMC2920013

[B26] Powles T Robinson D Stebbing J Shamash J Nelson M Gazzard B Mandelia S Moller H Bower M Highly active antiretroviral therapy and the incidence of non-AIDS-defining cancers in people with HIV infection J Clin Oncol 2009 14 884 890 10.1200/JCO.2008.19.6626 19114688

[B27] Hagiwara S Yotsumoto M Odawara T Ajisawa A Uehira T Nagai H Tanuma J Okada S Non-AIDS-defining hematological malignancies in HIV-infected patients: an epidemiological study in Japan AIDS 2013 14 279 283 10.1097/QAD.0b013e32835a5a7a 23014520

[B28] Chao C Leyden WA Xu L Horberg MA Klein D Towner WJ Quesenberry CP Jr Abrams DI Silverberg MJ Exposure to antiretroviral therapy and risk of cancer in HIV-infected persons AIDS 2012 14 2223 2231 10.1097/QAD.0b013e32835935b3 22951631 PMC4562666

[B29] Hishima T Oyaizu N Fujii T Tachikawa N Ajisawa A Negishi M Nakamura T Iwamoto A Hayashi Y Matsubara D Sasao Y Kimura S Kikuchi Y Teruya K Yasuoka A Oka S Saito K Mori S Funata N Sata T Katano H Decrease in Epstein-Barr virus-positive AIDS-related lymphoma in the era of highly active antiretroviral therapy Microbes Infect 2006 14 1301 1307 10.1016/j.micinf.2005.12.012 16697236

[B30] Levi F Lucchini F Negri E Boyle P La Vecchia C Trends in mortality from Hodgkin’s disease in Western and Eastern Europe Br J Cancer 2002 14 291 293 10.1038/sj.bjc.6600452 12177797 PMC2364217

[B31] Cox JA Lukande RL Nelson AM Mayanja-Kizza H Colebunders R Van Marck E Manabe YC An autopsy study describing causes of death and comparing clinico-pathological findings among hospitalized patients in Kampala, Uganda PLoS One 2012 14 e33685 10.1371/journal.pone.0033685 22432042 PMC3303855

